# A Backing-Layer-Shared Miniature Dual-Frequency Ultrasound Probe for Intravascular Ultrasound Imaging: In Vitro and Ex Vivo Validations

**DOI:** 10.3390/bios13110971

**Published:** 2023-11-06

**Authors:** Yashuo He, Xi Liu, Jiayi Zhang, Chang Peng

**Affiliations:** School of Biomedical Engineering, ShanghaiTech University, Shanghai 201210, China

**Keywords:** backing-layer-shared transducer, dual frequency, intravascular ultrasound, ultrasound imaging, ultrasound transducer

## Abstract

Intravascular ultrasound (IVUS) imaging has been extensively utilized to visualize atherosclerotic coronary artery diseases and to guide coronary interventions. To receive ultrasound signals within the vessel wall safely and effectively, miniaturized ultrasound transducers that meet the strict size constraints and have a simple manufacturing procedure are highly demanded. In this work, the first known IVUS probe that employs a backing-layer-shared dual-frequency structure and a single coaxial cable is introduced, featuring a small thickness and easy interconnection procedure. The dual-frequency transducer is designed to have center frequencies of 30 MHz and 80 MHz, and both have an aperture size of 0.5 mm × 0.5 mm. The total thickness of the dual-frequency transducer is less than 700 µm. In vitro phantom imaging and ex vivo porcine coronary artery imaging experiments are conducted. The low-frequency transducer achieves spatial resolutions of 40 µm axially and 321 µm laterally, while the high-frequency transducer exhibits axial and lateral resolutions of 17 µm and 247 µm, respectively. A bandpass filter is utilized to separate the ultrasound images. Combining in vitro phantom imaging analysis with ex vivo imaging validation, a comprehensive demonstration of the promising application of the proposed miniature ultrasound probe is established.

## 1. Introduction

When atherosclerotic plaques rupture within the coronary arteries, this becomes the primary trigger of coronary artery disease, a condition that can lead to severe complications such as acute coronary syndrome and sudden cardiac death [1,2]. Atherosclerosis is characterized by the accumulation of lipid-rich plaques on the arterial wall [3]. Plaques that have a high risk of rupture are known as vulnerable plaques, distinguished by a lipid-rich necrotic core covered by a thin-cap fibroatheroma (TCFA) [4]. Research indicates that TCFA serves as a precursor lesion to plaque rupture, with the thickness of the fibrous cap near the rupture site typically measuring approximately 23 ± 19 µm, generally less than 65 µm [5]. Consequently, high-resolution imaging techniques are necessary for the diagnosis of vulnerable plaques.

Intravascular ultrasound (IVUS) imaging technology utilizes miniaturized ultrasound transducers inserted into blood vessels to analyze the morphology of plaque tissue, allowing for the visualization of cross-sectional coronary artery walls and the quantitative assessment of lumen dimensions and plaque characteristics [6,7]. Traditional IVUS transducers typically provide axial resolutions ranging from 70 to 200 µm and lateral resolutions ranging from 200 to 400 µm [8]. However, the spatial resolution of conventional IVUS is inadequate for accurately measuring the thickness of thin fibrous caps. To address this limitation, optical coherence tomography (OCT) has emerged as a high-resolution imaging technique that utilizes backscattered infrared light, enabling the precise diagnosis of thin fibrous caps with a spatial resolution of approximately 10 µm [9]. Nevertheless, OCT has limited penetration depth in blood and vascular tissues, typically ranging from 1 to 2 mm [10,11]. The conventional IVUS transducers with center frequencies ranging from 20 to 40 MHz can provide an imaging depth from 5 to 10 mm [12]. To overcome the trade-off between imaging depth and resolution, the combination of IVUS and OCT imaging methods has been proposed [13,14,15]. However, integrating two imaging modalities increases the size of the catheter, presents challenges in registering IVUS and OCT images, reduces imaging speed, and incurs high production costs for the hybrid system [11,16].

A recent advancement in ultrasound imaging is the utilization of harmonic IVUS imaging [17]. This innovative technique takes advantage of the nonlinear response exhibited by contrast agents such as microbubbles to produce harmonic signals [18,19]. By harnessing these harmonics, imaging contrast and resolution can be greatly improved, providing valuable insights into vascular structure and pathological conditions [20,21,22]. Most contemporary clinical ultrasound systems employ second harmonic echoes to generate tissue harmonic images [23]. This approach necessitates the ultrasound transducer to transmit the fundamental ultrasound and receive the second harmonic induced by the transmitted ultrasound. Consequently, an ultrasound transducer with a −6 dB bandwidth greater than 70% is required [24]. However, fabricating miniature transducers with high center frequency presents challenges, having an impact on the optimal design process [25].

High-frequency IVUS imaging holds significant promise for enhancing arterial characterization due to its improved axial resolution, and it offers comparable penetration depth to that of OCT [26]. To be specific, an IVUS transducer with a center frequency of 80 MHz can provide an imaging depth of 2 mm [12]. To achieve a balance between imaging resolution and penetration depth, the concept of dual-frequency IVUS transducer has been introduced. This approach utilizes a lower frequency range (20–40 MHz) for sufficient penetration depth, while a higher frequency range (80–150 MHz) enables high-resolution imaging. Ma et al. [12] proposed three prototypes of dual-frequency IVUS probes with center frequencies of 35/90 MHz, 35/120 MHz, and 35/150 MHz. The study also presented three design schemes for the dual-frequency transducer: left-and-right, before-and-after, and back-to-back configurations. The left-and-right scheme resulted in a larger outer diameter of the catheter, approximately twice the size of a single-element transducer. Furthermore, the before-and-after scheme increased the front rigid length, reducing flexibility in navigating complex vessel structures. The back-to-back configuration was found to be preferable as it maximized space utilization and facilitated image co-registration by a simple 180° rotation.

Previous studies have demonstrated the advantages of multi-frequency ultrasound catheters, which provide both deep imaging capabilities and high-resolution imaging [12,27,28]. However, these studies often required individual coaxial cables to connect each transducer element, resulting in the need for multiple cables and an increased catheter diameter. Munding et al. [28] proposed a bidirectional dual-frequency IVUS probe in which the low-frequency transducers and high-frequency transducers were fabricated separately and then pressed together using a parallel press. Two micro-coaxial cables were utilized in this study to connect the dual-frequency transducers. Recently, Su et al. [29] developed an approach that uses a shared coaxial cable to connect two ultrasound transducers for dual-frequency imaging. A bandpass filter was utilized to separate signals from different transducers. The shared coaxial cable design reduced the size of the catheter and simplified the procedure associated with inserting coaxial cables into the catheter. It employed a single slip ring for efficient signal transmission and reception. However, this design needs a kerf in the backing layer to reserve space to connect the coaxial cable, which presents a certain complexity during the manufacturing process.

In this work, for the first time, we implemented a configuration where a low-frequency ultrasound transducer element (30 MHz) and a high-frequency ultrasound transducer element (80 MHz) were arranged back-to-back, sharing a single backing layer. This innovative design enabled the dual-frequency transducer to have an overall thickness of less than 700 µm. In addition, in order to simplify the interconnection of the dual-frequency transducer, a single coaxial cable was utilized, while a digital filter was employed to separate signals from each transducer element. We conducted in vitro phantom imaging and ex vivo porcine coronary artery imaging experiments to demonstrate the deep penetration capability of the low-frequency transducer and the high-resolution imaging of the lumen area achieved by the high-frequency transducer. These experimental results serve to validate the feasibility of the proposed dual-frequency IVUS probe in overcoming limitations associated with conventional IVUS catheters.

## 2. Materials and Methods

### 2.1. Transducer Design

**Figure 1:**
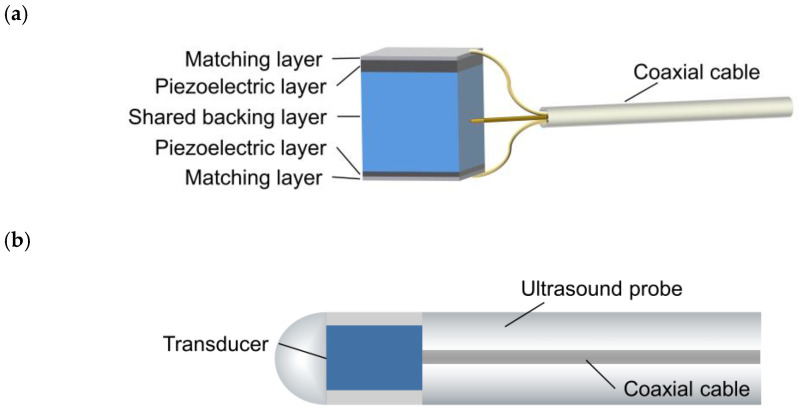
(**a**) Schematic diagram of the proposed backing-layer-shared dual-frequency ultrasound transducer. (**b**) Schematic diagram of the transducer integrated into an ultrasound probe.

The designed structure of the backing-layer-shared dual-frequency transducer is depicted in Figure 1a. This configuration enables the dual-frequency transducer to share a single backing layer, resulting in a reduced size of the ultrasound probe, as shown in Figure 1b. The back-to-back arrangement not only facilitates this structural design but also provides co-planar images of the blood vessel. The signal wire of a coaxial cable connects to the backing layer, while the ground wire of the coaxial cable is divided into two parts, connecting to the matching layers of the low-frequency and high-frequency transducers, respectively. The single coaxial cable serves the dual purpose of stimulating the dual-frequency transducer and receiving the return signal simultaneously. The shared backing layer and the use of a single coaxial cable contribute to minimizing the size of the ultrasound probe, making it suitable for comprehensive imaging of coronary artery blood structures.

The Krimholtz–Leedom–Matthaei (KLM) model is utilized to design the structure and size of the dual-frequency ultrasound transducer. The parameters of the proposed IVUS transducer are detailed in Table 1. The low-frequency transducer has a center frequency of 30 MHz, while the high-frequency transducer operates at 80 MHz. The aperture size of the dual-frequency transducer is 0.5 mm × 0.5 mm. PZT-5H is selected as the piezoelectric material for its excellent dielectric constant. For optimal electrical conductivity, a mixture of silver powder and epoxy is employed as the first matching layer. In order to provide an electrically insulating and water-resistant layer for the transducer, Parylene C with a thickness of 10 µm is vapor-deposited on the surface of the transducer, serving as both the second matching layer and protective coating. The entire thickness of the designed dual-frequency transducer is less than 700 µm.

### 2.2. Transducer Fabrication

**Figure 2:**
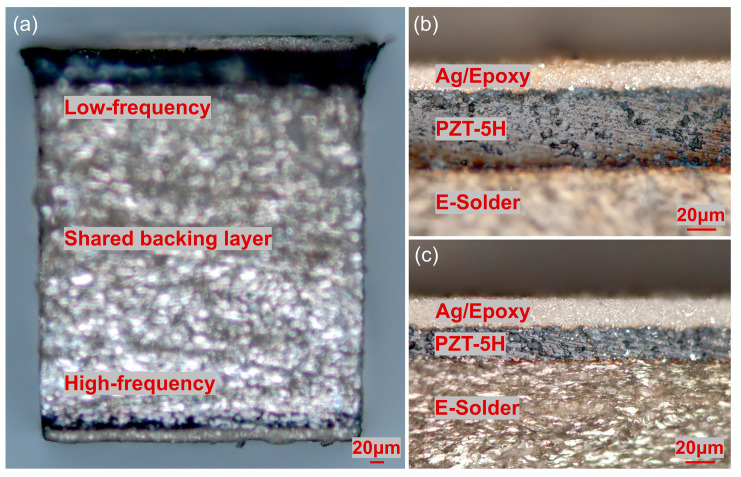
(**a**) Fabricated backing-layer-shared dual-frequency transducer. (**b**) The 30 MHz low-frequency transducer structure. (**c**) The 80 MHz high-frequency transducer structure.

The fabricated backing-layer-shared dual-frequency transducer is illustrated in Figure 2a. E-Solder 3022 (Von Roll USA Inc., Schenectady, NY, USA) was cured on a side of a piece of PZT-5H ceramic sample. Subsequently, the backing layer was lapped to a thickness of 500 µm, while the piezoelectric layer was lapped to 60 µm, serving as the low-frequency transducer. On the lapped side of the PZT-5H, a Cr/Au (500/1000 Å) electrode was sputtered. To create the first matching layer, a mixture of epoxy (Epo-Tek 301, Epoxy Technologies, Billerica, MA, USA) and silver particles with a diameter of 2–3 µm was utilized. The Ag/epoxy layer was lapped to a thickness of 20 µm. The structure of the 30 MHz transducer is depicted in Figure 2b. For the high-frequency transducer, the other piece of PZT-5H ceramic sample was connected to the lapped backing layer using E-Solder 3022. The piezoelectric layer was lapped to 25 µm, serving as the high-frequency transducer. Similarly, a Cr/Au (500/1000 Å) electrode was sputtered on the lapped side of the piezoelectric layer. Ag/epoxy was cured on the electrode and lapped to 20 µm to create the first matching layer. The structure of the 80 MHz transducer is illustrated in Figure 2c. After the above-mentioned fabrication processes, the dual-frequency transducer sample was diced into the designed aperture size, namely 0.5 mm × 0.5 mm. To establish electrical connections, E-Solder 3022 was employed to connect the signal wire of a coaxial cable to the backing layer. The ground wire of the coaxial cable was split into two parts, enabling connection to the matching layers of both the 30 MHz transducer and 80 MHz transducer, respectively. Finally, a thin layer of Parylene C was vapor-deposited on the surface of the dual-frequency ultrasound probe. This layer served as the second matching layer and also acted as a protective coating.

To compare the performance of the proposed backing-layer-shared dual-frequency transducer with that of single-element ultrasound transducers, two ultrasound transducers with center frequencies of 30 MHz and 80 MHz were fabricated as a comparison group, maintaining the same layer parameters and transducer size as the proposed probe.

### 2.3. Transducer Characterization

**Figure 3:**
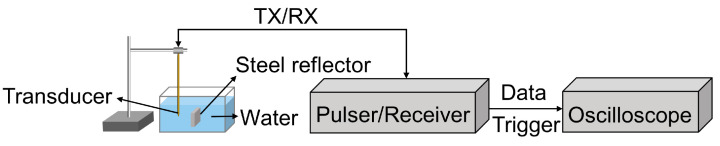
Pulse–echo response setup for the proposed dual-frequency transducer.

The piezoelectric and acoustic properties of the dual-frequency transducer were characterized. The dielectric property and electrical impedance of the dual-frequency transducer were assessed in air using an impedance analyzer (E4990A, Keysight Technologies, Santa Rosa, CA, USA). The experimental setup for the pulse–echo response is illustrated in Figure 3. The dual-frequency transducer was immersed in degassed water at room temperature. A steel reflector was employed as the target, allowing for the reliable and consistent reflection of the ultrasound waves. Excitation of the dual-frequency transducer was accomplished using a commercial pulser/receiver (DPR 500, JSR Ultrasonics, NY, USA), employing a 6.5 ns pulse width, 170 V pulse amplitude, and 2.5 μJ energy pulse. The echoes were efficiently captured and received by the same device. A digital oscilloscope (DSOX3054G, Keysight Technologies, USA) was utilized to acquire the echo signals.

### 2.4. Imaging Evaluation Setup

**Figure 4:**
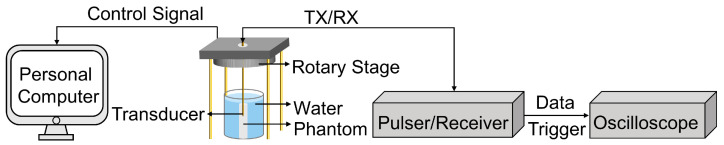
Imaging evaluation setup for the proposed dual-frequency transducer.

The imaging evaluation setup, as illustrated in Figure 4, was designed to assess the imaging performance of the proposed dual-frequency transducer. To facilitate the transducer excitation and amplification of the received signal, the proposed dual-frequency transducer was connected to a pulser/receiver, with the gain set to 40 dB and the pulse repetition frequency set to 200 Hz. The resulting echo signal was displayed on an oscilloscope, which was configured in the average mode to ensure accurate data acquisition. To facilitate rotational imaging, the ultrasound probe was mounted on a motorized precision rotary stage with an instantaneous rotation speed of 300°/min. This setup allowed for precise control over the rotation angle during the scanning process. A single pulse–echo response was recorded from the oscilloscope for every one-degree rotation of the ultrasound probe. To be specific, when the transducer was rotated by one degree using the rotary stage, the rotary stage was paused and the data were recorded from the oscilloscope. This process was repeated for each one-degree rotation, resulting in each image frame containing 360 lines. All the acquired data were processed using MATLAB to generate B-mode images after the scanning procedure was completed. By employing this comprehensive imaging evaluation setup, the performance and capabilities of the dual-frequency transducer could be thoroughly evaluated.

### 2.5. In Vitro and Ex Vivo Imaging

**Figure 5:**
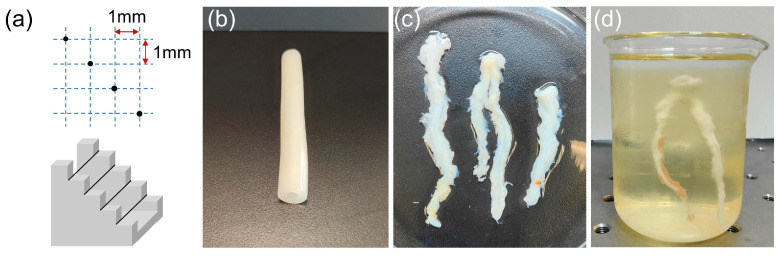
(**a**) Geometry of the wire phantom. (**b**) The finished blood vessel phantom released from the cylindrical mold. (**c**) Physical diagram of porcine coronary arteries. (**d**) A porcine coronary artery fixed by gelatin for ultrasound imaging.

In this work, a wire phantom was first fabricated to assess the spatial resolution of the proposed ultrasound probe, as shown in Figure 5a. The wire phantom consisted of four tungsten wires, with diameters of 10 µm, 20 µm, 30 µm, and 30 µm, arranged from left to right on a step mold. The axial and lateral distances between the two tungsten wires were both approximately 1 mm. The wire phantom was placed beneath the transducer, which was fixed on a precision motor. The wire phantom imaging scan was conducted by moving the transducer using the precision motor in a linear trajectory. This scanning process was performed perpendicular to the direction in which the wires were arranged.

A blood vessel phantom was then created to mimic the human coronary artery, with an inner diameter of 6 mm and a vessel wall thickness of 3 mm. Gelatin (Sinopharm Chemical Reagent Co., Ltd., Shanghai, China) was dissolved in thermal water at a weight ratio of 30% using a magnetic stirring heater. Sigmcell Cellulose (S3504, Sigma-Aldrich Ireland Ltd., Wicklow, Ireland) was added to the solution as scattering particles at a weight ratio of 3% [30]. The heating function was then turned off to allow the solution to cool to room temperature while continuous stirring prevented the settling of scattering particles. The gelatin solution containing gelatins and scattering particles was poured into a cylindrical mold. The mold consisted of a solid optical axis, a plastic straw, and two bearings. A solid optical axis with a diameter of 6 mm was inserted into one bearing and the plastic straw was attached to the outer circumference of the bearing. This arrangement created a cylindrical space between the solid optical axis and the plastic straw. The gelatin solution was then poured into this space. A heat gun was used to eliminate air bubbles from the mold [31]. The other bearing was then fixed on the opposite side of the mold to finalize the assembly. Subsequently, the mold was refrigerated for 6 h to complete the solidification process. Once cured, the vessel phantom was gently released from the mold using cold water as a lubricant. The image of the fabricated blood vessel phantom is shown in Figure 5b.

Ex vivo porcine coronary artery imaging was also conducted in this study. Figure 5c presented the physical diagram of the porcine coronary arteries (not diseased artery), providing a visual representation of their anatomy. In order to facilitate B-mode imaging, one of the porcine coronary arteries was fixed by gelatin, as depicted in Figure 5d.

## 3. Results

### 3.1. Performance of the Dual-Frequency Transducer

**Figure 6:**
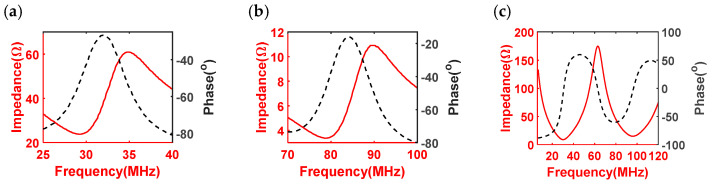
Electronical impedance magnitudes and phase angles of simulated (**a**) low-frequency transducer and (**b**) high-frequency transducer, as well as measured (**c**) proposed dual-frequency transducer.

Figure 6 illustrates the electrical impedance magnitudes and phase angles of both the simulated and measured values for the backing-layer-shared dual-frequency transducer. The simulated resonant frequencies for the low-frequency and high-frequency transducers were 29.4 MHz and 78.8 MHz, respectively (Figure 6a,b). Meanwhile, the measured resonant frequencies of the proposed dual-frequency transducer were 30.1 MHz and 97 MHz, respectively (Figure 6c). The measurement results closely align with the simulated results. However, it should be noted that the measured resonant frequency of the high-frequency transducer slightly exceeded the simulated value. This discrepancy could be attributed to the influence of the long coaxial cable with a length of 1.6 m, which introduced signal transition delay and loss, especially for higher-frequency signals. In addition, as the frequency increased, the phenomenon of signal attenuation became more pronounced, leading to a higher resonant frequency observed in the measurements. Consequently, the close agreement between the simulated and measured results validated the effectiveness of the proposed design and demonstrated the feasibility of the backing-layer-shared dual-frequency transducer.

**Figure 7:**
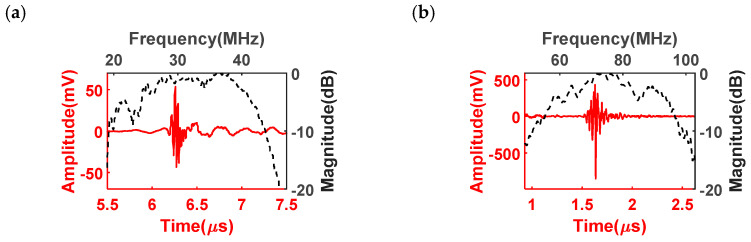
Pulse–echo responses of (**a**) 30 MHz low-frequency transducer and (**b**) 80 MHz high-frequency transducer.

The pulse–echo responses of the dual-frequency transducer were evaluated and illustrated in Figure 7. The low-frequency transducer was observed to possess a center frequency of 32 MHz, and the high-frequency transducer operated at a center frequency of 76 MHz. These observed frequencies closely align with the intended design values. A critical parameter characterizing the performance of ultrasound transducers is the −6 dB bandwidth, which represents the frequency range over which the transducer’s response remains within 6 dB of its maximum amplitude. In the case of the low-frequency transducer, an impressive −6 dB bandwidth of 62% was achieved, demonstrating its ability to effectively transmit and receive a wide range of frequencies. Similarly, the high-frequency transducer exhibited a bandwidth of 51%, indicating its capacity to handle higher frequencies while maintaining a significant portion of its maximum amplitude. The pulse–echo evaluation results are in agreement with the designed results. Importantly, the extensive bandwidths exhibited by the dual-frequency transducer indicate its remarkable potential for imaging application. The wide bandwidths offered by this transducer are of great significance in ultrasound imaging applications. A broader bandwidth allows for better differentiation and resolution of anatomical structures, resulting in improved image quality and diagnostic accuracy.

### 3.2. In Vitro Wire Phantom Imaging

**Figure 8:**
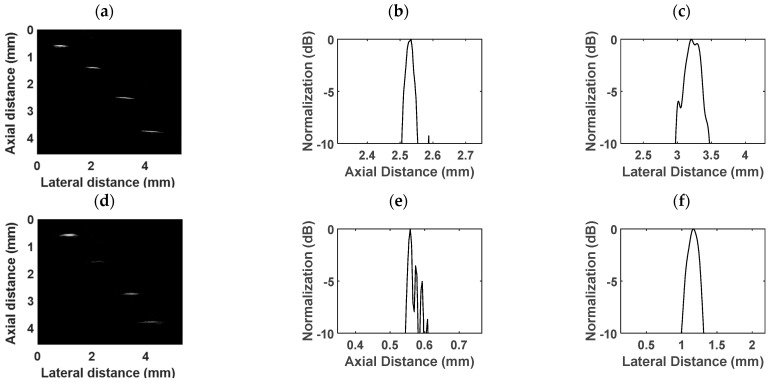
Wire phantom images acquired by the (**a**) low-frequency transducer and (**d**) high-frequency transducer. The (**b**) axial distance profile and (**c**) lateral distance profile of a wire obtained by the low-frequency transducer. The (**e**) axial distance profile and (**f**) lateral distance profile of a wire obtained by the high-frequency transducer.

The wire phantom images obtained with the proposed dual-frequency transducer are presented in Figure 8. The four tungsten wires have diameters of 10 µm, 20 µm, and two wires measuring 30 µm each, arranged from left to right. The dynamic range is 45 dB. Figure 8a displays the wire phantom imaging achieved using the low-frequency transducer, while Figure 8d illustrates the imaging obtained with the high-frequency transducer. The axial and lateral profiles are depicted in Figure 8b,c,e,f. In the case of the low-frequency transducer, the axial resolution was 40 µm, and the lateral resolution was 321 µm. For the high-frequency transducer, the axial and lateral resolutions were 17 µm and 247 µm, respectively.

The wire phantom images, along with the axial and lateral profiles, provide a comprehensive understanding of the resolution performance of the dual-frequency transducer. The enhanced axial resolution facilitates the identification of small abnormalities or lesions, while the improved lateral resolution enables the accurate differentiation of adjacent structures. The obtained results demonstrate that the low-frequency transducer effectively captures larger-scale features with satisfactory axial and lateral resolution. Conversely, the high-frequency transducer excels in imaging fine details with superior axial and lateral resolution, allowing for an enhanced visualization of intricate structures. Consequently, these features empower the dual-frequency transducer to provide valuable insights into the anatomical and pathological aspects of the imaged objects.

### 3.3. In Vitro Blood Vessel Phantom Imaging

**Figure 9:**
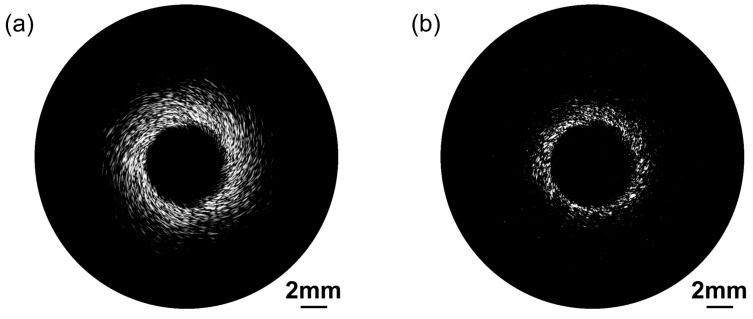
Blood vessel phantom ultrasonic images acquired by the proposed dual-frequency transducer. (**a**) Low-frequency transducer ultrasound imaging. (**b**) High-frequency transducer ultrasound imaging.

Figure 9 presents ultrasonic images of a blood vessel mimicking phantom behavior obtained using the proposed dual-frequency transducer. A bandpass filter was employed to separate signals from different frequency ranges. To achieve this, we applied the Kaiser window method for filter design. The filter parameters, including the order of the filter, normalized frequency band edges, and side lope suppression control was determined using the Kaiserord function. This approach allowed us to precisely isolate signals within the desired frequency range. The dynamic range is 50 dB. The ultrasonic image in Figure 9a acquired with the low-frequency transducer exemplifies its remarkable deep penetration capability by effectively visualizing the integrated blood vessel phantom. The spiral-like structure shown in Figure 9a was mainly due to the relatively low resolution of the 30 MHz ultrasound transducer for scattering particle imaging in the vessel phantom. Other reasons include the nonuniform distribution of scattering particles in the phantom and small shift in the transducer position during rotation. Figure 9b provides a higher level of detail and spatial resolution, achieved through the use of the high-frequency transducer.

The image acquired by the low-frequency transducer effectively captures the complete vessel structure. And the high-frequency transducer excels in providing high-resolution imaging, allowing for a detailed examination of surface features. Consequently, the blood vessel phantom images obtained using the proposed ultrasound probe provide compelling evidence of its deep penetration ability and high-resolution capability. This combination is crucial in clinical applications. The deep penetration capability of the low-frequency transducer facilitates the examination of deeper anatomical structures, and the high-resolution competence of the high-frequency transducer allows for the detection of subtle abnormalities and the visualization of intricate surface details.

### 3.4. Ex Vivo Porcine Coronary Artery Imaging

**Figure 10:**
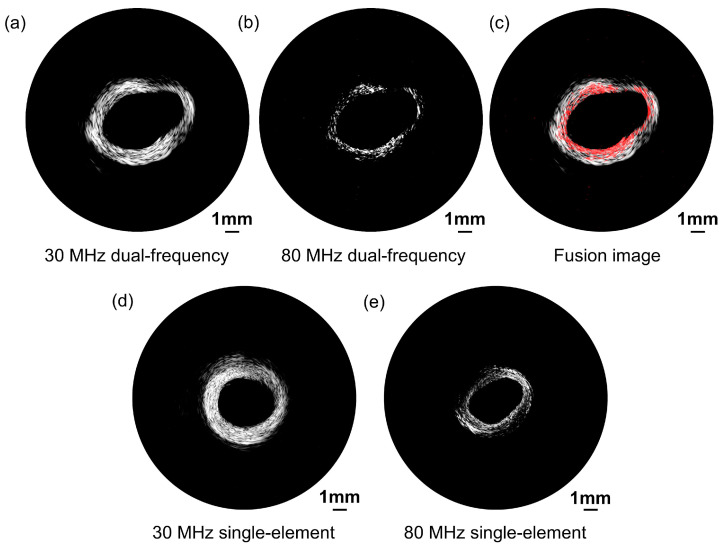
Porcine coronary artery ultrasonic images acquired by the proposed dual-frequency transducer. (**a**) Low-frequency transducer ultrasound imaging. (**b**) High-frequency transducer ultrasound imaging. (**c**) Fusion image combining low-frequency and high-frequency images together. Porcine coronary artery ultrasonic images acquired by the single-element transducers as the comparison group. (**d**) Low-frequency single-element transducer ultrasound imaging. (**e**) High-frequency single-element transducer ultrasound imaging.

Ex vivo porcine coronary artery images acquired by the proposed dual-frequency transducer are presented in Figure 10 with a 50 dB dynamic range. A bandpass filter was employed to distinguish signals from different frequency ranges. Figure 10a exhibits the low-frequency ultrasonic image, which portrays the entire porcine blood vessel. The image illustrates the remarkable capability of the transducer to penetrate deep into the tissue and effectively visualize the integrated structure of the blood vessel. This deep penetration ability is crucial when examining blood vessels located in complex tissue environments. In contrast, Figure 10b reveals the high-frequency ultrasonic image, which offers a more detailed representation of the vessel structure. Although it does not portray the integrated vessel structure, the high-frequency ultrasonic image provides a more detailed representation of the vessel structure. The high-resolution imaging capability of the high-frequency transducer enables the visualization of fine structural features and potential abnormalities with enhanced spatial resolution. A fusion image combining low-frequency and high-frequency images together is presented in Figure 10c.

Figure 10d,e present ultrasonic images of a porcine coronary artery obtained using single-element transducers, which serve as the comparison group. Please note that the images shown in Figure 10d,e were obtained from the same porcine vessel but represent a different cross-section compared with the images in Figure 10a,b. This variation is due to the differing positioning of the transducer in the vessel. The 30 MHz single-element transducer’s image reveals the complete blood vessel structure, while the 80 MHz single-element transducer provides a high-resolution image. However, the high-frequency transducer may be susceptible to increased attenuation, resulting in some areas appearing obscure. The imaging results from the comparison group are consistent with those obtained from the proposed dual-frequency transducer. Consequently, the proposed dual-frequency transducer effectively addresses the inherent tradeoff between imaging penetration depth and spatial resolution. The low-frequency transducer excels in penetrating deep into the tissue, enabling the visualization of the entire vessel structure. The high-frequency transducer prioritizes spatial resolution, providing a more detailed representation of the vessel surface. By combining both low and high frequencies, the proposed dual-frequency transducer offers a versatile solution that can adapt to the specific imaging requirements, as illustrated in Figure 10c.

## 4. Discussion

The utilization of micromachined ultrasound transducers in intravascular diagnosis and evaluation has been well established. Ultrahigh frequency transducers have emerged as a promising approach, offering exceptional imaging resolution comparable to the OCT imaging method. The practical implementation of a combined IVUS and OCT method presents challenges, primarily due to the complex structure and high production costs involved. To address these limitations, a dual-frequency transducer has been designed specifically for observing different situations within blood vessels. The integration of both low and high frequencies in an ultrasound catheter allows for a versatile imaging approach. The low-frequency transducer enables deep penetration, while the high-frequency transducer provides detailed information about the blood vessels. Nevertheless, the fabrication process of the dual-frequency transducer poses its own challenges. Manufacturing the two transducers individually and connecting each to a separate coaxial cable results in a relatively large probe thickness and a complex manufacturing process.

In this paper, we introduced the first known demonstration of a novel backing-layer-shared dual-frequency ultrasound probe with center frequencies of 30 MHz and 80 MHz. This innovative design offers several advantages over conventional transducers for intravascular ultrasound imaging applications. One key feature is the utilization of a single backing layer with a back-to-back structure, allowing for a compact thickness of less than 700 µm. This compact form factor is essential for applications where space constraints are a concern. Furthermore, the incorporation of a single coaxial cable to connect the transducer not only minimizes the overall size of the probe but also simplifies the fabrication process. This streamlined design reduces fabrication complexity and enhances the efficiency of manufacturing, enabling the cost-effective production of the dual-frequency transducer.

The proposed dual-frequency transducer featured a compact back-to-back structure with the size of 0.5 mm × 0.5 mm. This size is optimized for optimal performance and ease of integration into IVUS imaging systems. The compact dimensions make the transducer suitable for applications requiring high-resolution imaging in confined spaces. The probe was excited simultaneously, and the received images from different frequency ranges were separated using bandpass filters. The utilization of bandpass filters becomes particularly advantageous due to the significant disparity between the frequency spectra of 30 MHz and 80 MHz. This stark contrast in frequencies enables the filters to accurately isolate and separate the respective signals, thereby facilitating the distinct imaging of the different frequency ranges. By eliminating unwanted interference and isolating the desired frequency components, the bandpass filters enhance the clarity and quality of the received images.

The electrical impedance magnitudes illustrated the fact that the dual-frequency transducer exhibited resonant frequencies of 30.1 MHz and 97 MHz, respectively. The measured resonant frequency of the high-frequency transducer was slightly higher than the intended value, potentially attributed to signal transition delay and loss introduced by the long coaxial cable used in the setup. The higher frequency signals are more susceptible to attenuation, resulting in a higher resonant frequency being measured. The pulse–echo responses confirmed that the dual-frequency transducer had center frequencies of 32 MHz and 76 MHz, aligning with the designed values. The low-frequency transducer exhibited a −6 dB bandwidth of 62%, indicating its ability to effectively transmit and receive a wide range of frequencies. Similarly, the high-frequency transducer exhibited a bandwidth of 51%, highlighting its considerable potential for ultrasound imaging. The substantial bandwidths offered by the dual-frequency transducer indicate its capability to capture a broad spectrum of frequencies, which is crucial for achieving high-resolution and detailed ultrasound images.

In vitro wire phantom imaging was conducted in this study to assess the spatial resolution of the proposed ultrasound probe. The axial resolution for the low-frequency transducer was measured to be 40 µm, while the lateral resolution was determined to be 321 µm. For the high-frequency transducer, the axial resolution was found to be 17 µm, with a lateral resolution of 247 µm. The wire phantom imaging results exhibit a spatial resolution comparable to that of the previous dual-frequency transducer, thereby clearly demonstrating the high-resolution capability of the proposed transducer. To further evaluate the imaging performance, a blood vessel phantom was created to mimic a human blood vessel. The low-frequency transducer produced ultrasonic images that effectively depicted the overall structure of the blood vessel with its deep penetration capability. On the other hand, the high-frequency transducer exhibited a limited penetration depth of approximately 2 mm. While the imaging depth was constrained, it compensated for this by providing more detailed surface information of the phantom. This enhanced surface visualization enables a better characterization of surface structures, enhancing the overall diagnostic capabilities of the high-frequency transducer.

Moreover, ex vivo imaging of a porcine coronary artery was conducted to assess the performance of the transducer in a realistic situation. The low-frequency transducer illustrated its deep penetration ability by effectively visualizing the integrated blood vessel structure. The acquired images provided a comprehensive representation of the vessel morphology and enabled an accurate assessment of its overall structure. In contrast, the high-frequency transducer exhibited superior imaging resolution, providing clearer and more detailed information about the inner surface of the porcine blood vessel. This enhanced imaging resolution allows for a better visualization and characterization of subtle features, enabling the precise evaluation and diagnosis of potential abnormalities. The imaging of porcine coronary artery highlights the strengths and capabilities of the proposed dual-frequency ultrasound probe.

In our study, the implementation of a back-to-back configuration for the dual-frequency transducer, in conjunction with a signal processing strategy that employs filtering techniques, addresses potential interactions between the higher harmonics of the low frequency (30 MHz) and the fundamental frequency of the high frequency (80 MHz). The back-to-back configuration physically separates the transmission and reception of the low- and high-frequency signals, minimizing direct interactions between these frequency components. To further control these interactions, we utilize filters designed to isolate specific frequency components and attenuate undesired harmonics. This approach allows us to enhance the clarity and precision of imaging tissue structures. Consequently, the potential for interference between different frequency ranges is effectively managed, aligning with the imaging goals with the structure design and signal processing strategies.

In this study, we selected PZT-5H as the piezoelectric layer due to its outstanding stability. However, in comparison to single crystal materials such as PMN-PT and PIN-PMN-PT, PZT-5H exhibits a higher insertion loss. Furthermore, while the spatial resolution achieved with the proposed ultrasound transducer is of value, it falls short of matching the resolution capabilities of OCT. Nevertheless, our design offers a low-complexity and miniature approach for intravascular imaging compared to an OCT-IVUS hybrid system, which addresses the need for practical and accessible imaging solutions in certain clinical scenarios.

For the comparison group of single-element ultrasound transducers with center frequencies of 30 MHz and 80 MHz, in vitro wire phantom imaging experiments were conducted under identical experimental conditions to assess and compare the imaging resolution achieved by the different transducers. The results of these imaging experiments are presented in Table 2, providing a quantitative comparison of the imaging resolutions achieved by each transducer. The findings revealed that the proposed backing-layer-shared dual-frequency transducer exhibited comparable imaging resolution to the single-element ultrasound transducers. This indicates that the innovative design of the dual-frequency transducer, with its shared backing layer and compact size, did not compromise its imaging performance compared to the single-element counterparts.

## 5. Conclusions

This work is the first to present a backing-layer-shared dual-frequency ultrasound probe with a single coaxial cable interconnection for potential IVUS imaging applications. The proposed dual-frequency ultrasound probe not only features both high IVUS imaging resolution and deep penetration depth, but also fits the IVUS probe’s strict size constraints for clinical applications as well as reduces the interconnection complexity in IVUS probes. The dual-frequency ultrasound transducer was designed, fabricated, and characterized, which had center frequencies of 30 MHz and 80 MHz, respectively. The aperture size of both the low-frequency transducer element and the high-frequency element was 0.5 mm × 0.5 mm. The −6 dB bandwidth of the low-frequency transducer element and the high-frequency element was 62% and 51%, respectively. In vitro phantom imaging experiments demonstrated that the low-frequency transducer element achieved spatial resolutions of 40 µm axially and 321 µm laterally, while the high-frequency transducer element exhibited axial and lateral resolutions of 17 µm and 247 µm, respectively. Ex vivo porcine coronary artery imaging experiments validated the IVUS imaging capability of the developed probe. The quantitative results presented in this work provide a complete initial demonstration of future potential of the backing-layer-shared dual-frequency ultrasound probe. In the future, we anticipate that the backing-layer-shared dual-frequency ultrasound probe will open up new avenues for IVUS imaging applications. Further research directions include optimizing the probe’s design and exploring its potential for in vivo imaging to ensure its compatibility with clinical settings. By contributing to refine and develop the backing-layer-shared dual-frequency ultrasound probe, we aim to enhance its performance and expand its applicability, ultimately contributing to the advancement of IVUS imaging in clinical practice.

## Figures and Tables

**Table 1:** Parameters of the designed transducer.

Parameters	Low Frequency	High Frequency
Center frequency (MHz)	30	80
Aperture size (mm)	0.5 × 0.5	0.5 × 0.5
Piezoelectric material	PZT-5H	PZT-5H
Thickness (µm)	60	25
First matching layer material	Ag/epoxy	Ag/epoxy
Thickness (µm)	20	20
Second matching layer material	Parylene C	Parylene C
Thickness (µm)	10	10
Backing layer material	E-Solder 3022	E-Solder 3022
Thickness (µm)	500	500

**Table 2:** Comparison of the different transducers.

	Center Frequency	Axial Resolution	Lateral Resolution
Proposed transducer	30 MHz	40 µm	321 µm
	80 MHz	17 µm	247 µm
Single element	30 MHz	50 µm	349 µm
	80 MHz	12 µm	182 µm

## Data Availability

Not applicable.
